# Morphology Control of Monomer–Polymer Hybrid Electron Acceptor for Bulk-Heterojunction Solar Cell Based on P3HT and Ti-Alkoxide with Ladder Polymer

**DOI:** 10.3390/ma15031195

**Published:** 2022-02-04

**Authors:** Yasuyuki Ueda, Yuki Kurokawa, Kei Nishii, Hideyuki Kanematsu, Tadashi Fukumoto, Takehito Kato

**Affiliations:** 1 Department of Materials Chemistry and Bioengineering, National Institute of Technology, Oyama College, 771 Nakakuki, Oyama 323-0806, Japan; 2 Graduate School of Life Science and Systems Engineering, Kyushu Institute of Technology, 2-4 Hibikino, Wakamatsu-ku, Kitakyushu-shi 808-0196, Japan; kurokawa.yuki207@mail.kyutech.jp; 3 Department of Materials Science and Engineering, National Institute of Technology, Suzuka College, Shiroko-cho, Suzuka 510-0294, Japan; kanemats@mse.suzuka-ct.ac.jp; 4 Technical Research Institute, Nishimatsu Construction Co., Ltd., 1-17-1 Minato-ku, Toranomon, Tokyo 105-6490, Japan; tadashi_fukumoto@nishimatsu.co.jp; 5 NPO Energy Education, 6-20-21 Ekiminami, Oyama 323-0822, Japan

**Keywords:** solar cell, energy conversion, organic–inorganic hybrid material, Ti-alkoxide, P3HT

## Abstract

We report the morphology control of a nano-phase-separated structure in the photoactive layer (power generation layer) of organic–inorganic hybrid thin-film solar cells to develop highly functional electronic devices for societal applications. Organic and inorganic–organic hybrid bulk heterojunction solar cells offer several advantages, including low manufacturing costs, light weight, mechanical flexibility, and a potential to be recycled because they can be fabricated by coating them on substrates, such as films. In this study, by incorporating the carrier manager ladder polymer BBL as the third component in a conventional two-component power generation layer consisting of P3HT—the conventional polythiophene derivative and titanium alkoxide—we demonstrate that the phase-separated structure of bulk heterojunction solar cells can be controlled. Accordingly, we developed a discontinuous phase-separated structure suitable for charge transport, obtaining an energy conversion efficiency higher than that of the conventional two-component power generation layer. Titanium alkoxide is an electron acceptor and absorbs light with a wavelength lower than 500 nm. It is highly sensitive to LED light sources, including those used in homes and offices. A conversion efficiency of 4.02% under a 1000 lx LED light source was achieved. Hence, high-performance organic–inorganic hybrid bulk heterojunction solar cells with this three-component system can be used in indoor photovoltaic systems.

## 1. Introduction

In recent years, multiple coated solar cells have been proposed; these include dye-sensitized solar cells [1,2,3,4], organic thin-film solar cells, and perovskite solar cells, all of which continue to improve in terms of efficiency and functionality [5,6,7,8,9,10,11,12,13,14,15,16,17,18]. In particular, the power conversion efficiency (PCE) of perovskite solar cells has exceeded 20%, and high-performance electronic devices based on organic–inorganic hybrid materials have been demonstrated [18]. A PCE of 15% has been reported for single-junction organic thin-film solar cells [19], and a further performance improvement is expected as they move towards practical use. The theoretical PCE of organic thin-film solar cells has exceeded 20% [20], and further improvements in efficiency are desirable. The general photoactive layer (power generation layer) of organic thin-film solar cells is composed of p-type semiconductor material as an electron donor and n-type semiconductor material as an electron acceptor. Fullerene derivatives, such as 6,6-phenyl-C61 or C71-butyric acid methyl ester (PCBM_60_ or PCBM_70_), have been used as typical n-type semiconductor materials for electron acceptors. However, fullerene derivatives have low air stability due to oxidation, when the inks of these derivatives are formulated for the fabrication of the power generation layer [21]. Therefore, the development of air-stable electron acceptors is desired. Previous studies from this perspective have reported organic–inorganic hybrid thin-film solar cells that use metal alkoxides and oxides as electron acceptors [22,23]. Among metal oxides and alkoxides, titanium dioxide and alkoxide are light absorbers that can absorb light below 500 nm, making them highly sensitive to LED light sources used indoors and suitable for indoor photovoltaic systems. However, when titanium alkoxide is used as an electron acceptor, the PCE is only 0.03%, and the short-circuit current density (J_sc_) is 191 μA/cm^2^ [22]. Therefore, to achieve the higher efficiency required for practical use, it is necessary to improve the J_sc_. The thickness of the bulk heteroelectric layer is of the order of approximately 100 nm (97–102 nm), and the ultra-thin film is collectively responsible for light absorption, charge separation, and charge transport [22]. Therefore, it is important to control the morphology, considering all aspects of charge separation, charge transport, and optical absorption [5,6]. Recently, many p-type semiconducting polymers with optical absorption in the long-wavelength range have been developed to obtain a higher J_sc_ [8,9], but their power generation characteristics are inadequate. One of the reasons why sufficient improvements in efficiency are not observed is that it is difficult to maintain solubility in solvent and control the self-assembly (self-aggregation) of a polymer to form a phase-separated structure that is suitable for charge separation and charge transport.

We propose a phase-separated structure for the photoactive layer by utilizing the steric hindrance of the electron-accepting material and demonstrate its effectiveness [22,23]. However, these methods, based on monomers with electron-accepting properties, are insufficient to efficiently suppress the self-assembly of polymers as electron donors. This is because the self-assembly capacity of polymers is higher than the self-assembly capacity of monomers. In contrast, organic thin-film solar cells with bulk heterostructures that use polymers with electron acceptor properties have been reported. However, the formation of dense phase-separated structures that are necessary for the construction of many charge-separated interfaces is not sufficient due to the self-aggregation of the electron-donor and electron-acceptor polymers [24]. This report details our attempt to control the phase-separated structure in the power generation layer by using both monomers and polymers, which have different self-aggregation and molecular hindrance properties, as electron acceptors. In this report, p-type semiconducting polymeric compounds were used as the electron donor and titanium alkoxide was used as the monomer electron acceptor, and poly(benzimidazobenzophenanthroline) (BBL) as the polymer electron acceptor [25]. To create a co-continuous charge transport path in the acceptor, BBL, known to be a ladder polymer with strong self-aggregation, was employed as the electron acceptor [25,26]. It is worth noting that charge separation has been confirmed for each electron acceptor in combination with the p-type semiconducting polymer compound poly(3-hexylthiophene-2,5-diyl) (P3HT) [27]. By controlling the phase-separated structure of the three-component bulk heterostructures using monomer–polymer composite electron acceptors, we achieved high-performance charge management in bulk heterojunction thin-film solar cells.

## 2. Materials and Methods

General Procedures and Materials

**Figure 1:**
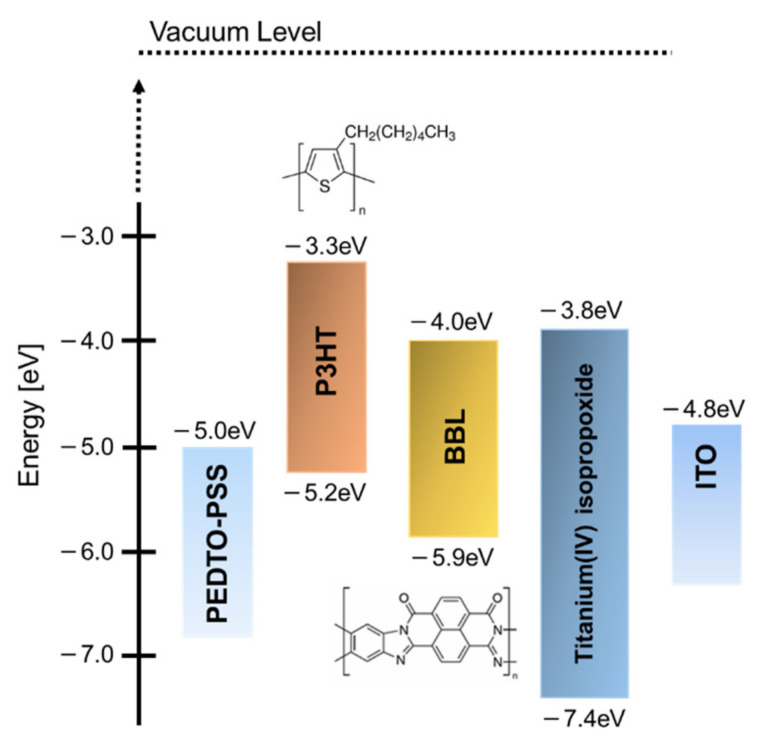
Materials and energy diagrams.

For the photoactive layer, we used P3HT as the electron-donor material, titanium(IV) isopropoxide as the monomeric electron-acceptor material, and BBL as the polymeric electron-acceptor material. These were purchased from Sigma-Aldrich Japan (Tokyo, Japan). Figure 1 shows the molecular structures and energy diagrams of each material.

The solar cells were first fabricated by patterning them on an indium tin oxide (ITO)-coated glass substrate (15 Ω per square). These substrates were washed with detergent, acetone, and isopropyl alcohol for 15 min each and then dried in air. In addition, the surface was treated with a UV/O_3_ cleaner (Filgen, Model UV253E, Aichi, Japan) for 20 min. For the electron collection layer, a functional thin-film layer was fabricated by dropping 0.50 wt.% titanium(IV) isopropoxide dissolved in isopropyl alcohol onto a substrate and spin coating at 2000 rpm for 30 s on an ITO substrate [23]. Next, a chlorobenzene solution of P3HT (3.0 wt.%) A and chlorobenzene solution of titanium(IV) isopropoxide (6.0 wt.%) B were prepared separately [28].

**Figure 2:**
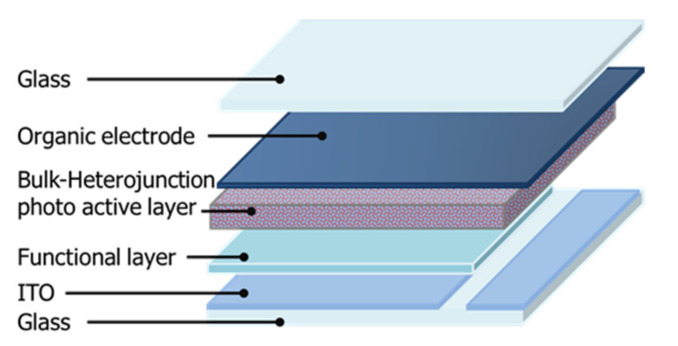
Device structure for solar cells.

These solutions were mixed (A+B = 1:1 wt.%) to prepare an ink for forming a charge management layer in the single electron-accepting system. This was spin coated on the functional layer at 2000 to 3500 rpm for 60 s. The functional layers were about 50 nm thick. The charge management layer of the monomer–polymer hybrid electron-accepting system was also fabricated through the same process. For the n-type photoactive layer solution, a solution of P3HT (3.0 wt.%) was added to chlorobenzene (electron-donor solution), and a solution of titanium(IV) alkoxide (6.0 wt.%), methanesulfonic acid (1.2 wt.%), and BBL (0.5, 0.1, 0.15, 2.0, and 3.0 wt.%) dissolved in chlorobenzene was prepared. Each solution was prepared by mixing (1:1 wt.%). These charge management layers were dried in the dark in air and at room temperature for 10 min, after which the thickness of each photoactive layer was about 50 nm. Furthermore, to promote the hydrolysis of titanium(IV) isopropoxide, the substrate containing the photoactive layer was placed in a humidity chamber overnight at 80% humidity. Organic electrodes (50 μm) were applied using a screen printer (Mitani Micronics, MEC-2400, Tokyo, Japan). Poly(3,4-ethylenedioxythiophene)–poly(styrenesulfonic acid) (PEDOT-PSS, Clevios S V3, Leverkusen, Germany) was purchased from Baytron (Tokyo, Japan) and used to form the organic electrodes. Finally, the photoactive layer and organic electrodes were laminated with epoxy resin. Figure 2 shows the structure of the device. The current density–voltage (J–V) characteristics of the solar cells were measured under AM 1.5G quasi-solar radiation (San-Ei Electric, XES-40S1, Osaka, Japan) at 100 mW/cm^2^ using a DC voltage and current source/monitor. The incident photon-to-current efficiency (IPCE) of the device was measured using the DC voltage and current source/monitor (Bunko-Keiki Co., Ltd., CEP-2000RS, Tokyo, Japan). The light intensity was corrected using a silicon photodiode reference cell (Bunko-Keiki Co., Ltd., BS-520, Tokyo, Japan). To evaluate the photovoltaic performance, an LED light source (Bunko-Keiki Co., Ltd., BLD-100, Tokyo, Japan) was used at 1000 lx to simulate standard indoor light. The phase-separated structure of the photoactive layer was observed using a scanning electron microscope (SEM, JEOL, JSM-7800, Tokyo, Japan). The UV–Vis/near-IR spectrum of each photoactive layer obtained by spin coating on the glass substrate was measured using a UV spectrophotometer (Shimadzu, UV-1800, Kyoto, Japan). Furthermore, the electrical conductivity of each photoactive layer on the spin-coated glass substrate was measured by the four-point probe method using a resistivity system (Mitsubishi Chemical Analytech Co., Ltd., Lorester-GX MCP-T700, Kanagawa, Japan) [29,30,31,32,33].

## 3. Results

**Figure 3:**
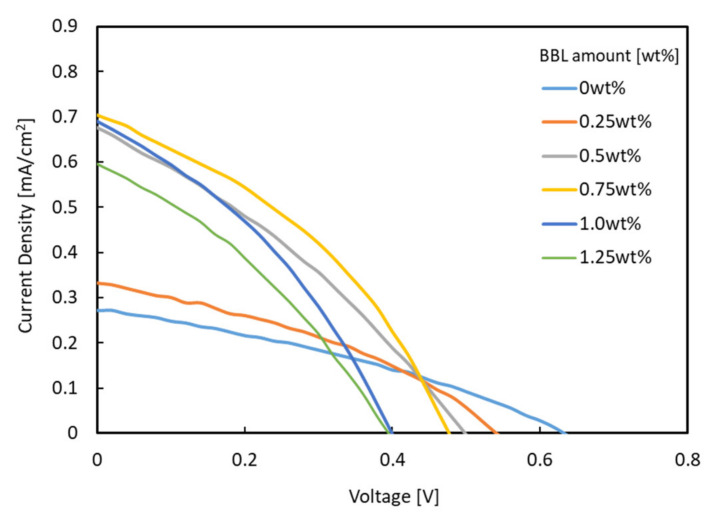
J–V characteristics for solar cells under AM 1.5.

Figure 3 shows the J–V characteristics of each bulk heterojunction solar cell using titanium-alkoxide and P3HT as the photoactive layer. Table 1 lists the corresponding performance parameters.

**Figure 4:**
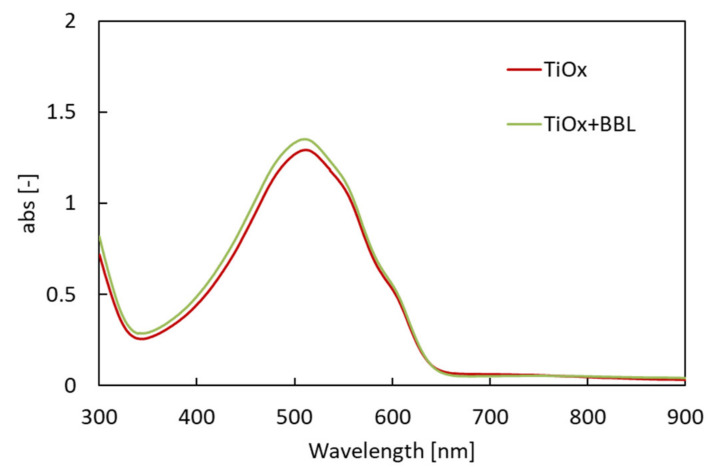
UV–Vis/near-IR spectrum for photoactive layers.

When a monomer–polymer hybrid electron acceptor was used in the charge management layer, the J_sc_ value was higher than that of the device using only titanium alkoxide as a single electron acceptor. In particular, the device with a BBL of 0.75 wt.% showed the highest J_sc_ (0.704 mA/cm^2^). The relationship between the amount of BBL added and the J_sc_ value is discussed below. Most of the hybrid electron-accepting devices showed higher fill factor (FF) values than the Ti electron-accepting devices. In contrast, the use of the hybrid electron acceptor reduced the open-circuit voltage (V_oc_) from 0.632 V to a minimum of 0.451 V. As the lowest unoccupied molecular orbital (LUMO) of BBL is lower than the LUMO of Ti-alkoxide, V_oc_ decreased when BBL was added. As shown in Table 1, the solar cell energy conversion efficiency increased from 0.057% to a maximum of 0.126%, while improving the J_sc_ and FF values. The UV–Vis/near-IR spectra (optical absorption spectra) of each photoactive layer were measured to verify these results. Figure 4 shows the UV–Vis/near-IR spectrum measurement results.

**Figure 5:**
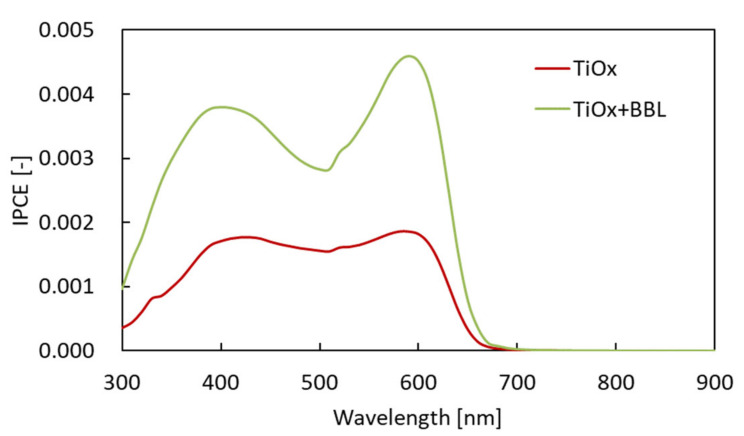
IPCE spectra of each solar cell (red: solar cell with single electron acceptor (TiOx only); green: solar cell with hybrid electron acceptor (TiOx + BBL)).

The results show the optical absorption spectrum of each charge management layer to be almost the same, with a peak at 515 nm. Therefore, the improvement in J_sc_ was not affected by the increase in the light absorption of the charge management layer. Furthermore, the addition of BBL does not affect the light absorption of the charge management layer. Next, the IPCE of each solar cell (red: solar cell with single electron acceptor (TiOx only); green: solar cell with hybrid electron acceptor (TiOx + BBL)) was investigated. Figure 5 shows the IPCE spectra [34]. 

**Figure 6:**
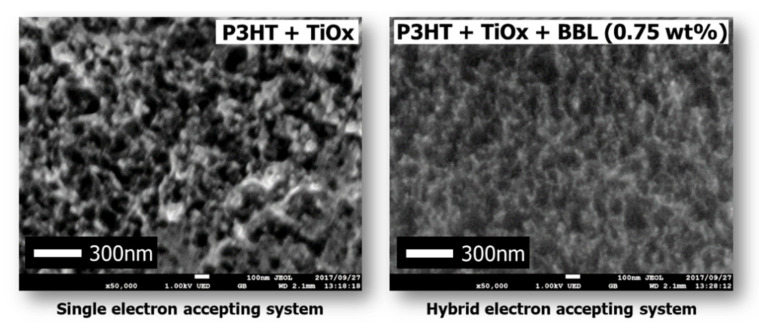
SEM images of photoactive layers.

The IPCE of the solar cell with the hybrid electron acceptor was higher than the IPCE with the Ti-alkoxide single electron acceptor. Therefore, in this case, it was found that the high value of J_sc_ in the cell using the hybrid electron acceptor influences the morphology of the charge management layer. There are three main aspects of the morphology of the charge management layer that influence the achievement of a high J_sc_. First, it is vital that the excitons generated by photoabsorption diffuse from the electron-donor polymer to the p/n interface. Next, it is important to express charge separation in order to generate a large number of free carriers at the p/n interface. Finally, after charge separation, a charge management layer is required to move the deactivated free carriers from the p/n interface to the electrodes at high speed, without exciting them. In other words, the charge management layer requires the construction of a large number of p/n interfaces for charge separation and a continuous phase-separated structure for the transport of free carriers. Several studies have already reported that control of the phase-separated structure is a critical factor in obtaining high J_sc_ values in bulk heterojunction solar cells [5,6,7,8,9,22,23,24]. To investigate the reason for the improvement in IPCE and J_sc_, charge separation and charge transport were studied by SEM for each phase-separated structure of the charge management layer [35,36,37]. Figure 6 shows an SEM image of the charge management layers. The results show that, in comparison with the conventional single electron-acceptor system, the monomer–polymer hybrid electron acceptor dramatically improves the titanium(IV) isopropoxide network for carrier transport, without reducing the extent of the p/n interface. Preferential self-assembly of the polymer as an electron donor is inhibited by the chemical bulk performance of the monomer as an electron acceptor and the self-assembled polymer. The electron-donor p-type semiconducting polymer has a long chain and is sterically bulky, forming a large structure that affects the photoactive layer. In addition, the conventional electron acceptor was only a monomer. Therefore, the self-assembly of the electron acceptor was not preferential with respect to that of the electron donor. Accordingly, the formation of the electron-donor phase becomes dominant, and the electron-acceptor phase is formed following the electron-donor phase structure.

**Figure 7:**
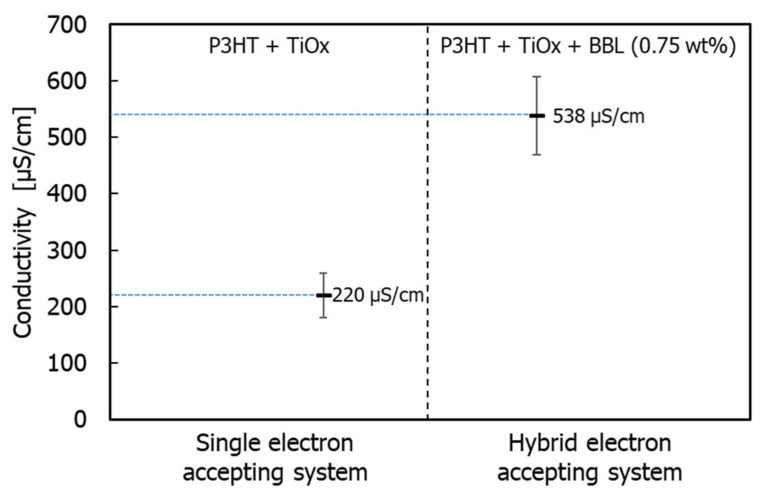
Individual value of electrical conductivity of photoactive layers.

**Figure 8:**
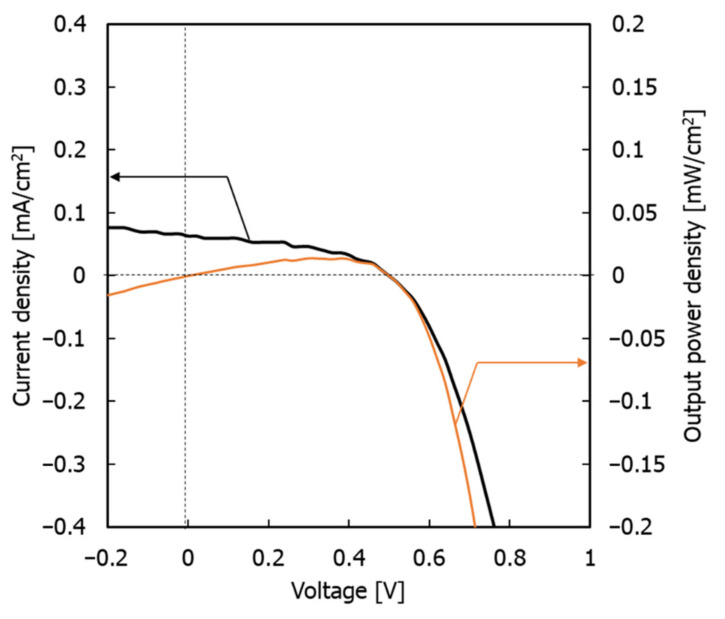
J–V characteristics for solar cell under the LED indoor light at 1000 lx.

Therefore, in conventional single electron-acceptor systems, phase formation occurs between polymer phases, and the structure of the electron acceptor becomes coarse and irregular. However, in this monomer–polymer hybrid electron-accepting system, the preferential phase formation of the electron-donor polymer is prevented by the electron acceptor. As a result, the electron donor and acceptor can each form a continuous phase-separated structure due to their individual self-assembly faculties. This phenomenon is also demonstrated by the individual evaluation of the electrical conductivity of the charge management layer in the presence of BBL. Figure 7 shows the measured electrical conductivity of the charge management layer [30,31]. The results show that the addition of BBL significantly improves electrical conductivity and the carrier transport network in the thin films. As the electron-accepting polymers are ladder polymers with high self-assembly and template-forming faculties, the phase of the titanium(IV) isopropoxide monomer followed the ladder structure of BBL [25,26]. Accordingly, the peak increase in J_sc_ occurs at 0.75 wt.% of the BBL addition, which means that the charge separation interface decreases due to the delay in the construction of the template network. Thus, compared with the conventional titanium(IV) isopropoxide system alone, titanium(IV) isopropoxide as an electron acceptor significantly improves the charge transport network, owing to its high electron transport capacity. In other words, the enhanced electronic charge transport efficiency of the titanium(IV) isopropoxide carrier management network resulted in high J_sc_ values, without affecting light absorption. Finally, the performance of the device was measured under a standard room light of 1000 lx. Figure 8 shows the J–V characteristics.

The maximum power density (P_max_), J_sc_, FF, and PCE were 14.07 µW cm^−2^, 0.063 mA cm^−2^, 0.5 V, 44.46%, and 4.02%, respectively. These results demonstrate that the system has the potential to be used as an indoor light-based power generation system [38].

## 4. Conclusions

In this study, using a hybrid electron acceptor based on the BBL template formation mechanism, the power generation layer was templated into a co-continuous and finely phase-separated structure. This improved the charge transport capacity without decreasing the charge separation efficiency, and increased J_sc_ by approximately 2.2 times. The improvements in the morphology were confirmed by SEM images of the power generation layer (photoactive layer). This confirmed that the hydrolyzed titanium(IV) isopropoxide phase with high electron transport capacity could construct a continuous network supported by the BBL template. The power generation layer has high photosensitivity to LED light sources, and the characteristics under a 1000 lx LED light source showed a high photoelectric conversion efficiency of 4.02%. This demonstrated the potential of using the device as an indoor power source. In contrast, the addition of BBL was found to decrease V_oc_. This is because the LUMO level of BBL is smaller than that of titanium(IV) isopropoxide, and when using it as a composite material to form the power generation layer, the apparent LUMO of the electron acceptor was lowered. Therefore, to realize higher energy conversion performance and efficiency, it is necessary to search for materials with a high LUMO level and a high template formation function, characterized by the self-assembly capacity.

## Figures and Tables

**Table 1:** Solar cell performance under AM 1.5.

Category	BBL Amount [wt%]	J_sc_ [mA/cm^2^]	V_oc_ [V]	Fill Factor	PCE [%]
Single electron acceptor	0	0.272	0.632	0.336	0.057
	0.25	0.332	0.540	0.364	0.065
	0.5	0.676	0.498	0.318	0.107
Hybrid electron acceptor	0.75	0.704	0.476	0.376	0.126
	1.00	0.689	0.399	0.350	0.096
	1.25	0.596	0.395	0.330	0.078

## Data Availability

The data presented in this study are available in insert article here.
